# Asset mapping score analysis: a novel public health research methodology applied to maternal and child health resources in New Orleans

**DOI:** 10.1186/s13690-023-01042-1

**Published:** 2023-02-28

**Authors:** Jack Healy, Shokufeh Ramirez, Megan Knapp, Carolyn Johnson

**Affiliations:** 1 Center of Excellence in Maternal and Child Health, Tulane University School of Public Health and Tropical Medicine, New Orleans, LA USA; 2 Texas Christian University School of Medicine, TCU Box 297085, Fort Worth, TX 76129 USA; 3 Department of Public Health Sciences, Xavier University of Louisiana, New Orleans, LA USA

## Abstract

**Background:**

Asset mapping is a commonly used method in public health to identify and describe the resources within a community. However, there is currently a lack of standardization in the methods used for asset mapping, which can make it difficult for users to apply the method and compare results between different studies. In this article, we present a new approach called Asset Mapping Score Analysis (AMSA), which is a framework for collecting and organizing data on community assets. We provide an example of the AMSA method through its application in the evaluation of maternal and child health resources in New Orleans, Louisiana.

**Results:**

The AMSA approach consists of five steps and results in a data collection tool that uses a scoring system to quantify the functional and content areas defined by the users. This method is flexible, reproducible, quantitative, inexpensive, and can be adapted to fit the needs of different geographic areas and fields of study. It can also be repeated over time to monitor changes in systems. We conducted a pilot study to examine the participation of local maternal and child health organizations in four functional areas (education, direct services, policy/advocacy, and research) and 22 content areas.

**Conclusions:**

In addition to describing the AMSA method and providing an example of its application, we also discuss the methodological issues involved in using the AMSA approach. These include considerations related to study design, data analysis, and interpreting results. We assess the strengths, limitations, and potential future directions of the AMSA method. Finally, we present the results of our AMSA study on maternal and child health organizations in New Orleans to illustrate the utility of this approach. Our findings suggest that the AMSA method is a valuable tool for understanding and characterizing the assets and resources within a community.

## Background

Community asset mapping is a methodological tool that is commonly deployed in research and program planning in diverse fields, including public health, behavioral sciences, community development, social work, and governmental services [1–3]. Asset mapping is the process of systematically observing and describing resources available within a community, including both tangible and intangible resources [4–6]. Organizations and individuals can use data from asset maps to promote collaboration and make informed decisions based on current resources, build on strengths, identify potential partners, and better distribute limited resources [7, 8]. Asset mapping shares characteristics with the more commonly used approach of needs mapping, which has long been used to characterize deficits in resources in specific locations [9, 10]. Like needs mapping, asset mapping can be used to study social phenomena in a defined geography, involves participatory research, and can be adapted to answer a variety of research questions or meet project goals [11]. However, unlike needs mapping – which focuses on gaps, deficiencies, and shortages - asset mapping focuses on the resources, strengths, capacities, and talents of a population, rather than emphasizing its inadequacies [12, 13].

Although asset mapping techniques have been utilized for decades across multiple disciplines, a standardized methodology has yet to be established. Standardization of the asset mapping approach would offer a robust framework for researchers, public health officials, or community development leaders to initiate their own asset mapping projects. Additionally, it allows comparability between asset mapping projects studying similar topics in different regions, as well as monitoring of the changes in systems over time by applying the same method to the same geographic location longitudinally. In this article, the traditional asset mapping process is expanded by adding a novel scoring component to assess, quantify, and better understand the resources of a community. A step-by-step guide is provided to describe the procedures.

Although the primary purpose of this article is to describe the new methodology, the authors also provide an example of its usage. This AMSA methodology was used to evaluate Maternal and Child Health (MCH) resources in New Orleans, Louisiana, United States. MCH outcomes in Louisiana and New Orleans are poor relative to other states and cities within the United States [14]. In 2020, Louisiana had the second highest infant mortality rates (7.5 infant deaths per 1000 live births) in the United States [15]. In 2013, the prevalence of low birth weight in New Orleans was 13.4% compared to a national prevalence of 6.0% and a state prevalence of 11.0% [16]. Additionally, a report examining child mortality in Louisiana during the period of 2015–2017, found that Louisiana ranked in the top ten states for highest infant and child mortality in almost every age group [17]. Furthermore, in 2020, the teen birth rate (defined as number of births per 1000 females aged 15 to 19 years old) was 29.1, compared to the national rate of 18.8, making Louisiana the fourth highest state in country for teen birth rates [18].

It is well known that MCH outcomes are not evenly distributed [19, 20]. Key social determinants of health, including race and racism, poverty, and differences in power and opportunity have massive impacts on every aspect that influences maternal and child health [21]. For example, a 2010 study by Bryant and colleagues reported that compared to White women, Black women experience higher rates of fetal demise, preterm birth, and maternal mortality [22]. Additionally, a report by the Louisiana Department of Health found that in 2017, the rate of pregnancy-related deaths was 5.6 times higher in Black women than White women [23]. That same study found that non-White women had increased risk of maternal diabetes and obesity. These racial inequities in maternal and child health are not isolated to the perinatal period, and unfortunately persist for years. From 2015 to 2017, the relative risk of death in a Black infant aged 0 to 1 years-old in in New Orleans was 2.2 compared to White infants [17]. Another example of health inequity is poverty, which plays a key role in mediating MCH outcomes. An analysis from 2019 found that of the more than 50,000 uninsured children in Louisiana, the largest share was made up of children from low-income families [24]. Given these poor health outcomes and marked health inequities, maternal and child health programming in New Orleans is worthy of study, and warrants nuanced investigation. Asset mapping score analysis provides a method for doing so.

Research and clinical practice suggest that many outcomes related to maternal and infant morbidity and mortality, as well as reproductive and sexual health, are associated with community-level factors and resources [25]. Environmental factors such as access to healthcare and early intervention services and availability of resources to meet daily needs influence maternal and child health outcomes [26]. Better understanding the role and scope of MCH organizations in New Orleans, their client reach, and their specific job functions, may help in strategic planning to improve MCH outcomes by identifying the strengths and weaknesses of the local MCH system and leveraging existing resources [27].

The purpose of this work was to describe a new research methodology, called Asset Mapping Score Analysis (AMSA). This method can be used to answer several types of research questions, or characterize and describe complex social phenomena. The AMSA method is conducive for partnership and collaboration, and allows for input from multiple stakeholders, including local leaders, academic subject experts, government officials, and other members and key stakeholders in the community. These individuals and groups can be incorporated in almost every phase of the study from study design and inception up to data analysis and dissemination of the results. AMSA is flexible, inexpensive, easy to reproduce, and can provide rich qualitative and quantitative data. This article describes the steps of the AMSA methodology so that this novel framework can be applied to different topical areas and in different geographic locations. Finally, this methodology was applied to study New Orleans MCH organizations as an example of its usage and the results of that study are presented.

## Methods

### Overview

**Fig. 1:**
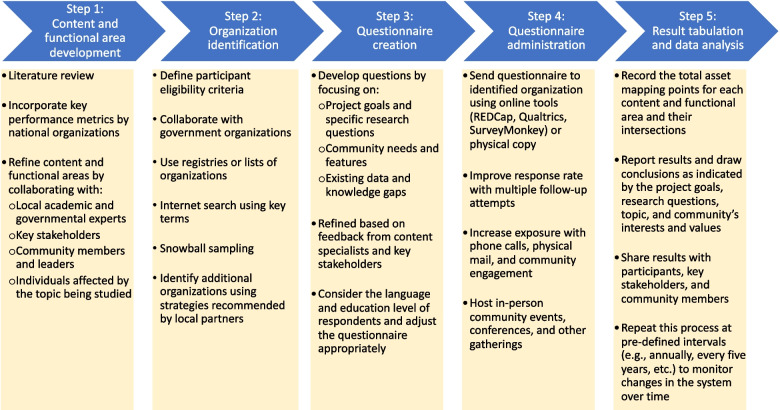
Asset Map Score Analysis (AMSA) Study Design

Asset Mapping Score Analysis is conducted in five steps (Fig. 1). First, the study team determines the content and functional areas to study by reviewing the existing data and knowledge gaps. In the second step, an initial list of organizations to be surveyed is compiled. Additional organizations can be added to the study as it proceeds and new organizations are identified. Third, the questionnaire or any other data collection forms are developed and revised. The fourth step is data collection, which involves contacting organizations and inviting them to participate in the study by completing the questionnaire and collecting any other data as indicated by the study topic and population. Finally, the data is tabulated to calculate the sum of all Asset Mapping Points for each functional and content area. The following sub-sections will describe each step in more detail and also provide information about how these steps were collected for the study of MCH organizations in New Orleans.

### Step 1: content and functional area development

The first step in AMSA is to develop content and functional areas to evaluate, which serve as the primary variables analyzed in this method. Functional areas are broader categories of the domain of work performed, whereas the content areas are more specific topics. AMSA studies will likely have fewer functional areas than content areas. This step can be completed by conducting a thorough review of the literature on the topic, accessing local and national key performance metrics, and refining topics by soliciting input and feedback from community leaders, key stakeholders, academic experts, and government officials.

For the study of MCH organizations in New Orleans, after completion of a literature review, content areas and functional areas were derived using three major sources: 1) the American Academy of Pediatrics (AAP) *Bright Futures* outcomes, 2) the national performance measures from the Health Resources and Services Administration Maternal and Child Health Bureau (HRSA MCHB); and 3) establishing and collaborating with a working group that included MCH academic staff at Tulane University, local government officials, and New Orleans MCH community leaders and key stakeholders [28, 29]. Content areas were finalized by consolidating similar measures from each source and, to focus on the goals of this project more closely, removing measures that had only a direct clinical component.

### Step 2: identifying organizations

The next step in AMSA requires determining which organizations to invite to participate in the study. To do this, researchers can use pre-existing lists or registries of related organizations that work in that field. Study staff can also search the internet using relevant key terms to identify additional organizations. If a working group was formed in the first step, the research team could also inquire with members of the group about organizations who may be eligible. Finally, snowball sampling methods can be employed where responding organizations are allowed to recommend additional organizations. During this stage, the study team also identifies eligibility and exclusion criteria based on desired outcomes.

For the New Orleans MCH study, this step was completed using four sources. First, study staff utilized a list of current and former partners of the Tulane Center of Excellence in Maternal and Child Health of organizations. Second, a local government organization provided a list of their partner organizations related to MCH. Third, an extensive online search was conducted to find any additional organizations not included via the first two sources. Search terms included content areas of interest listed below along with the term “New Orleans” (e.g., “breastfeeding New Orleans”). Results from these three sources were combined, duplicate organizations were removed, and an initial list of organizations was created. The fourth method for identifying organizations was snowball sampling. Online questionnaires were delivered as described below, and respondents had the option to recommend additional organizations to participate. Organizations discovered via snowball sampling who met the eligibility criteria were invited to participate in the study.

To be eligible for inclusion in the sampling frame, an organization had to be involved directly or indirectly in work related to the health of women, children, and/or families. Organizations were excluded if they were 1) exclusively educational institutions (e.g., K-12 schools), 2) exclusively provided medical care (e.g., hospitals, clinics, pharmacies, outpatient rehabilitation services, etc.), or 3) exclusively involved in childcare (e.g., preschool or daycare). Additionally, any organization that self-identified as not working in the MCH field was excluded.

### Step 3: questionnaire development

The third step in the AMSA approach is the development of the questionnaire that will be used to collect data, most likely a questionnaire or survey. The questionnaire will ask all participating organizations to identify the functional and content areas in which they work. Additional questions can be added, and these questions will be determined based on the goals and specific research questions of the study, the needs of the community and its unique features, existing data, and knowledge gaps. Before being administered to the organizations, the questionnaire can be reviewed by the partnered working group and refined based on their feedback. Study staff should make intentional efforts to consider the language and education level of respondents and adjust the questionnaire appropriately. The New Orleans MCH study staff created a questionnaire that inquired about the functional and content areas developed in Step 2. The questionnaire also included other questions, including the type of organization (public/government, not-for-profit, for-profit), the number of employees, and the population(s) the organization serves. Before administering to participating organizations, the questionnaire was reviewed by the working group. A pilot test was then conducted with a small sample. The questionnaire was then refined and finalized using feedback from this process.

### Step 4: Questionnaire Administration

The next step in the AMSA method requires contacting organizations and disseminating the data collection tools. This can be done with multiple approaches, including administering the questionnaire via email, phone, or physical mail. Multiple attempts should be made and different individuals at the organization may need to be contacted.

For the MCH study, all listed organizations with a publicly available email address were sent an invitation via REDCap to participate in the study by completing the questionnaire [30]. When possible, email addresses of administrative staff within the organization were found and used for questionnaire distribution. If the first contacted individual did not respond, additional employees were emailed. When organizations were not responsive, phone calls were made. For organizations that did not have a website with staff email addresses or a phone number, study staff attempted to contact the organization via social media (e.g., sending a message to the organization’s official Facebook page using the same standardized script). Contact was made with no more than three persons per organization with no more than five attempts total.

### Step 5: result tabulation and data analysis

The final step in this method is analyzing the data and sharing the results. To do so, the total asset mapping points for each content and functional area and their intersections should be calculated and summarized. Then, the results can be reported and conclusions can be drawn as indicated by the project goals, research questions, topic, and community’s interests and values. The results can then be shared with participating organizations, community members, and the working group. This process can be repeated at pre-defined intervals (e.g., annually, every 5 years, etc.) to monitor changes in the system over time.

For the New Orleans MCH study, questionnaire data were tabulated into a matrix with the 4 functional areas as the columns and the 22 content areas as rows, resulting in 88 total cells. Questionnaire respondents were asked to indicate all the content and functional areas that their organization works in (non-mutually exclusive), resulting in a binary yes/no result. These data were used to calculate an Asset Mapping Score for each functional and content area. Indicating “Yes” to any given cell contributed 1 point to that functional area and that content area. For example, if a respondent answered “Yes” to breastfeeding research, that contributed 1 point to the breastfeeding content area and 1 point to the research functional area. The total score for each functional area was the sum of all asset mapping points from all content areas across all organizations. Similarly, the total score for each content area was the sum of all asset mapping points from all functional areas across all organizations. Therefore, each of the four functional areas had a total asset mapping score, and each of the 22 content areas had a total asset mapping score.

## Results

**Fig. 2:**
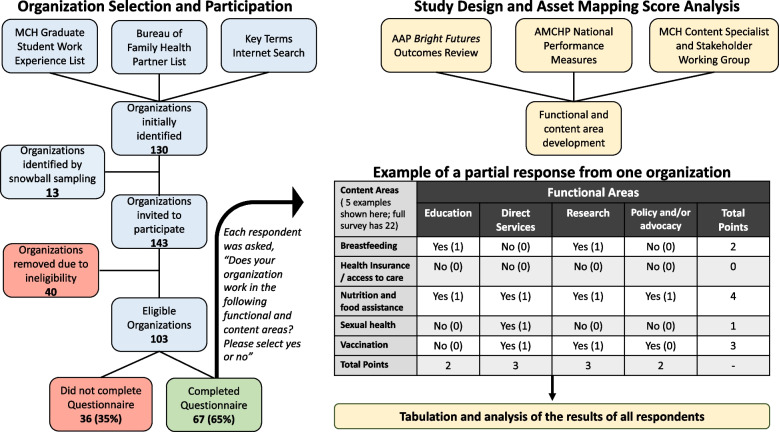
Study design and execution for Asset Mapping Score Analysis (AMSA) of maternal and child health organizations in New Orleans. The left side of the process diagram shows how organizations were selected and how many participated in the study. The right side shows how the questionnaire was developed to determine the functional and content areas. It also provides a partial example of how a respondent may answer the questionnaire. The diagram shows all four functional areas (education, direct services, research, and policy and/or advocacy), and five of twenty-two total content areas. The results of all responses were tabulated to produce the final dataset. MCH= Maternal and Child Health. AAP=American Academy of Pediatrics. AMCHP= Association of Maternal & Child Health Programs

**Fig. 3:**
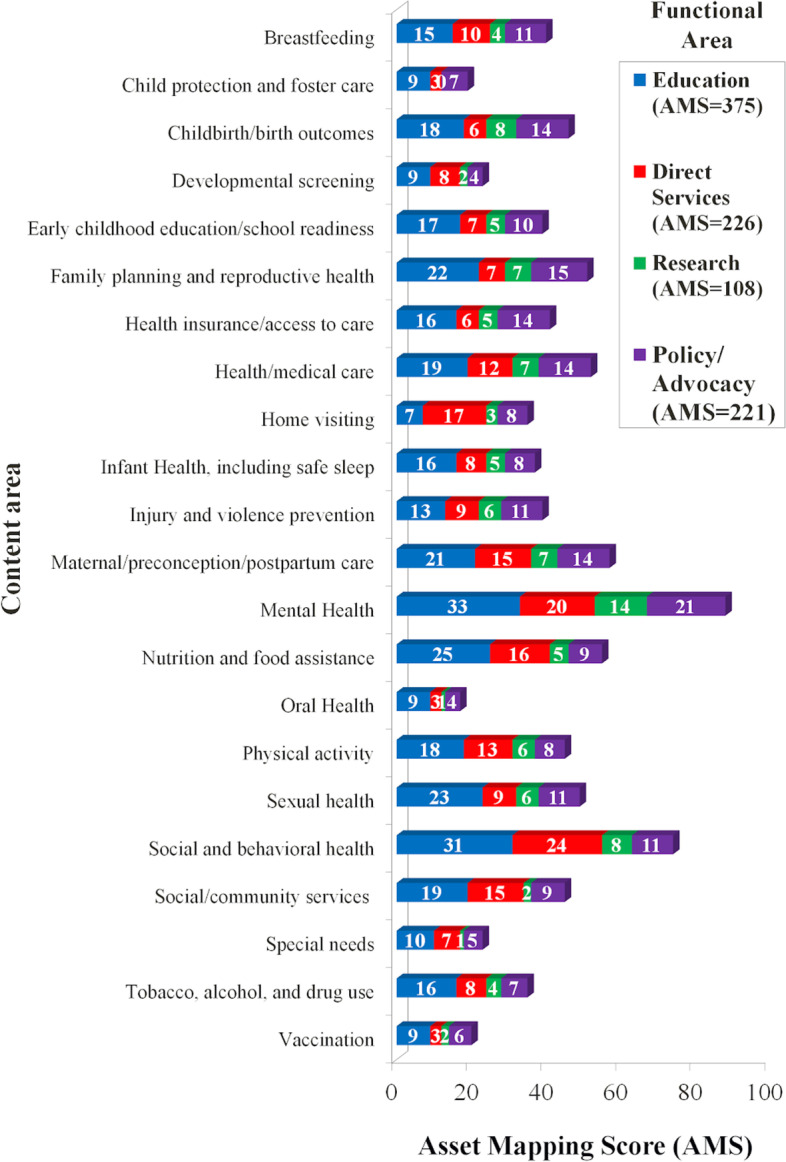
New Orleans maternal and child health organizations work tabulated using the Asset Mapping Score Analysis (AMSA) framework. Each organization was asked if they perform work (yes/no) in specific content areas (rows) and in four functional areas: education (blue), direct services (red), research (green), and policy/advocacy (purple). A content area received one Asset Mapping point per functional area for each organization that responded yes. For example, if one organization responded yes for breastfeeding education and research, it contributed two points towards the Asset Mapping Score for breastfeeding. The total AMS for the functional areas is seen is the figure legend, and the total AMS for each content area is displayed at the end of its row

The five steps of this project using the AMSA method to study MCH organizations in New Orleans were conducted from March 2017 to May 2018. The workflow and results of this study are summarized in Fig. 2. In the first step, study staff developed a total of four functional areas and twenty-two content areas by utilizing the AAP *Bright Futures* outcomes*,* HRSA MCHB National Performance Standards, and recommendations from the local MCH working group. The functional areas were: 1) education, 2) direct services, 3) policy/advocacy, and 4) research. The 22 content areas are listed in Table 1 and Fig. 3.

**Table 1:** Functional and content areas of New Orleans Maternal and Child Health organizations

Content area	Functional area (number of organizations)				
	Education	Direct Services	Research	Policy and/ or Advocacy	Total Points
Breastfeeding	15	10	4	11	**40**
Child protection and foster care	9	3	0	7	**19**
Childbirth/birth outcomes	18	6	8	14	**46**
Developmental screening	9	8	2	4	**23**
Early childhood education/school readiness	17	7	5	10	**39**
Family planning and reproductive health	22	7	7	15	**51**
Health insurance/access to care	16	6	5	14	**41**
Health/medical care	19	12	7	14	**52**
Home visiting	7	17	3	8	**35**
Infant Health, including safe sleep	16	8	5	8	**37**
Injury and violence prevention	13	9	6	11	**39**
Maternal/preconception/postpartum care	21	15	7	14	**57**
Mental Health	33	20	14	21	**88**
Nutrition and food assistance	25	16	5	9	**55**
Oral Health	9	3	1	4	**17**
Physical activity	18	13	6	8	**45**
Sexual health	23	9	6	11	**49**
Social and behavioral health	31	24	8	11	**74**
Social/community services (including legal and financial assistance)	19	15	2	9	**45**
Special needs	10	7	1	5	**23**
Tobacco, alcohol, and drug use	16	8	4	7	**35**
Vaccination	9	3	2	6	**20**
**Total Points**	**375**	**226**	**108**	**221**	**930**

In step two, using the first three sampling methods, an initial list of 130 organizations was generated. An additional 13 organizations were identified using snowball sampling. This lead to a total of 143 organizations invited to participate. Of those, 40 did not meet the eligibility criteria, yielding a final list of 103 organizations. Sixty-seven organizations completed the questionnaire (65.1% response rate).

Table 1 provides the complete matrix for the AMSA tabulation. These data are also visually represented in Fig. 2. The total points for each functional area were as follows: Education = 375, Direct services = 226, Research = 108, and Policy/Advocacy = 221. The content areas with the most points were mental health (88), social and behavioral health (74), and maternal/preconception/postpartum care (57). The content areas with the fewest points were oral health (AMS = 17), child protection or foster care (AMS = 19), and vaccination (AMS = 20). These data are also represented visually in Fig. 3.

## Discussion

The purpose of this article is to explain a novel method for conducting research at the community level, using a new approach called Asset Mapping Score Analysis (AMSA). The AMSA design was used to assess MCH organizations in New Orleans and the results of that study are presented here. Researchers and public health officials can apply this new methodology to other topics in different geographic locations. Asset mapping approaches have existed for almost 30 years, and have been employed in multiple countries, in regions ranging from rural to urban, and applied to a diverse array of topics in public health, medicine, social work, economics, and other social and behavioral sciences [31–40]. Asset mapping methods have unique benefits and offer distinct advantages that needs mapping alone cannot provide [41, 42]. Despite the longevity and utility of this tool, there have been few attempts to develop structured and reproducible protocols for conducting asset mapping studies. The purpose of this project is to describe a new method that others can use to conduct their own asset mapping studies.

A recent scoping review by Luo and colleagues provides a thorough summary of the origins and evolution of asset mapping along with several example studies [3]. They assert that there is a large variation in the methodological approaches and rigor of asset mapping studies. In their review, they found 28 articles that used an asset mapping methodology to conduct public health research. They found that little has been done to standardize asset mapping approaches, and public health projects that have applied this methodology have great variability in their methodologies and protocols utilized to collect information, as well as the processes to interpret this data. Other authors have identified additional weaknesses in asset mapping studies, which include the lack of unified definitions for “community assets” or “health assets”, time and cost barriers associated with mapping projects that are resource- or personnel-intensive, and concerns that asset mapping projects that rely on responses from individuals may misrepresent the values or views of the broader community [2, 8, 40].

As such, a well-defined and reproducible method, such as the AMSA approach, may be useful to provide a robust tool that can be used to strengthen the quality of real-world evidence collected by asset mapping projects. Our AMSA tool addresses these weaknesses by using a novel framework in which both functional areas (e.g., education, direct services, research, and policy/advocacy) and content areas are evaluated. This provides richer data that may be used in combination with outcomes data, survey data, or other results to provide a holistic knowledge base of the strengths, weaknesses, opportunities, and threats of the New Orleans MCH field. Furthermore, this novel approach may supplement other forms of data to provide insights that can be used to inform policy recommendations, program planning and budgeting, or new research questions for MCH organizations. Additionally, it addresses the concerns of methodological weakness identified by previous authors by allowing users to clearly define what health assets they are evaluating, provides a low-cost and quick method, and allows for characterization of organizations and institutions.

This analysis found that the MCH functional area with the most asset mapping points was education (375 points), and the one with the least was research (108 points). The content areas with the most asset mapping points were mental health, social and behavioral health, and maternal/preconception/postpartum care. The content areas with the fewest asset mapping points were oral health, child protection or foster care, and vaccination. While MCH outcomes have been well-studied and documented in New Orleans, there is a knowledge gap in recording which organizations are doing what work. This may result in inefficiencies where several organizations are working on the same problems but are not collaborating. Asset mapping may therefore reduce these redundancies and help organizations work together on similar efforts. Additionally, such mapping can identify (mis-)alignment between assets and resources.

This methodology has several strengths. First, by differentiating functional areas from service areas, resources can be described and quantified in novel ways. For example, to the authors’ knowledge, no one has ever previously characterized or quantified MCH organizations in New Orleans allowing for a more comprehensive assessment of the MCH resources available in the area. Second, AMSA is highly flexible. Users could change the functional and content areas under study to better suite their project goals. Third, AMSA is inexpensive and can be used to study complicated topics even in resource-limited settings. The largest costs associated with this study were staff wages and technologic resources. It is reasonable to conclude that this approach could be scaled up to study larger samples in greater geographic areas at a relatively low cost. Fourth, AMSA data can be combined with other methods, like needs mapping, survey studies, and financial or economic studies. Finally, the AMSA study can be repeated over time to detect changes in the communities longitudinally.

This method also has key limitations. Most importantly, AMSA data is not a direct measure of outcomes. For example, in this assessment of MCH resources in New Orleans, the content area with the most asset mapping points was mental health. However, it is difficult to determine if the high number of points is because there is a robust mental health support system in the study population, or if it reflects a significant burden of mental health morbidity. Likewise, the number of asset mapping points for any content area is a measure of the total number of organizations working in that content area among all four functional areas, and therefore, does not reflect the volume of work being done by a single organization. For example, a content area with a low number of points, such as vaccination, may be handled by only a few organizations, but those organizations may have a high degree of coverage. Furthermore, some consideration must be given to the fact that points were determined by organization self-report. While self-report was important because it allowed organizations to select all the work that organizations do in functional or content areas, it is also possible that differences in interpretation or definitions may have affected these results. Finally, the response rate for our questionnaire was 65.1%. It is difficult to determine how the findings of this project may have changed with a higher response rate.

The AMSA method can be applied to other geographic locations and fields of study as a tool to better understanding the work being done in those contexts. The information obtained via this methodology can be combined with the other data from traditional models to expand the body of knowledge. Other researchers, program planners, or service providers could use a similar framework to describe the functional areas and content areas that are relevant in their field and adapt the methods to suit their needs in terms of describing the pertinent strengths or weaknesses unique to their situation. Furthermore, because this framework is flexible, future users can focus on the components that are most important to them. Other users may add factors such as the organizations’ budgets, eligibility criteria, number of clients reached per unit time, partner organizations, or geographic information. Future work may find the AMSA method to be a valuable tool in other contexts because it provides information that needs mapping and outcomes research cannot. It is flexible and can incorporate quantitative, qualitative, and geographic data, and can be easily modified to meet the specific needs of the users. Given the strengths of this method, including its relative low-cost, flexibility, adaptability, and standardization that allows for comparability, we recommend use the use of the AMSA method for individuals or organizations interested in asset mapping.

## Data Availability

Raw data may be available from the authors upon reasonable request and with permission of the Tulane Center of Excellence in Maternal and Child Health.
